# A Novel Missense Variant of the *ABCD1* Gene in X‐Linked Adrenoleukodystrophy in Chinese Family

**DOI:** 10.1002/mgg3.70148

**Published:** 2025-10-03

**Authors:** Hongxia Fu, Lu Han, Xianhong Liu, Bin He, Pei He, Junjian Hu

**Affiliations:** ^1^ Department of Neurology, SSL, Central Hospital of Dongguan City Affiliated Dongguan Shilong People's Hospital of Guangdong Medical University Dongguan China; ^2^ Department of Central Laboratory, SSL, Central Hospital of Dongguan City Affiliated Dongguan Shilong People's Hospital of Guangdong Medical University Dongguan China; ^3^ The Center for Precision Medicine, SSL, Central Hospital of Dongguan City Affiliated Dongguan Shilong People's Hospital of Guangdong Medical University Dongguan China; ^4^ Department of Obstetrics and Gynaecology, The Center for Reproductive Medicine, Nanfang Hospital Southern Medical University Guangzhou China

**Keywords:** *ABCD1*, novel mutation, VLCFA, X‐ALD

## Abstract

**Background:**

We identified a novel ABCD1 variant (c.773T>G, p.Leu258Arg, NM_000033.4) in a Chinese pedigree affected by X‐linked adrenoleukodystrophy (X‐ALD). This missense variant in exon 1 is predicted to be pathogenic and likely constitutes the genetic basis of the disease phenotype in this family.

**Methods:**

*ABCD1* gene sequencing was performed in the Chinese pedigree. The pathogenicity of identified variants was assessed using computational prediction tools. Subcellular localization studies were conducted, and very‐long‐chain fatty acid (VLCFA) levels were quantified in patient‐derived samples.

**Results:**

Sequencing analysis identified a hemizygous missense variant in the *ABCD1* gene (c.773T>G; p.Leu258Arg). In silico pathogenicity prediction using SIFT and PolyPhen‐2 algorithms classified the p.Leu258Arg substitution as deleterious. Functional characterization revealed that the p.Leu258Arg variant impairs the peroxisomal membrane localization of the ABCD1 protein. Consistent with the established role of *ABCD1* in peroxisomal β‐oxidation, individuals harboring this variant exhibited significantly elevated serum levels of VLCFA. Specifically, the C26:0/C22:0 ratio was elevated 2.8‐fold compared to control values, confirming impaired VLCFA metabolism.

**Conclusion:**

In accordance with the “Standards and Guidelines for the Interpretation of Sequence Variants” established by the American College of Medical Genetics and Genomics (ACMG), we assessed the pathogenicity of the novel *ABCD1* gene variant c.773T>G. This variant meets the following ACMG evidence criteria: PM1 (located within a critical functional domain or mutational hotspot known to lack benign variation); PM2 (absent or observed at very low frequency in population databases e.g., gnomAD, EXAC, 1000 Genomes); PP3 (multiple in silico prediction tools consistently suggest a deleterious effect on the gene or gene product). Integrating this evidence (PM1 + PM2 + PP3), the variant is classified as likely pathogenic based on ACMG guidelines. Experimental data from this study further substantiate the pathogenicity of the c.773T>G variant located in exon 1 of the *ABCD1* gene. This finding broadens the spectrum of known pathogenic mutations in *ABCD1* associated with X‐ALD and provides crucial information for the molecular diagnosis of affected patients.

## Graphical abstract

This study identifies a novel ABCD1 gene variant (c.773T>G, p.Leu258Arg) linked to X‐linked adrenoleukodystrophy in a Chinese family, demonstrating its pathogenic impact via disrupted peroxisomal localization and impaired fatty acid metabolism, thus expanding the mutation spectrum and advancing molecular diagnosis and familial genetic counseling.

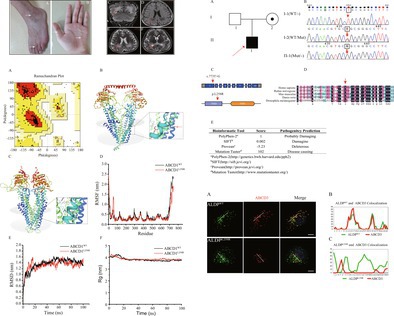

## 1 Introduction

X‐linked adrenoleukodystrophy (X‐ALD, OMIM: 300100) is a devastating inherited disorder predominantly affecting males, characterized by progressive demyelination and axonal degeneration in the nervous system and adrenal insufficiency. With a global prevalence of approximately 1 in 17,000 newborns (Mukherjee et al. 2024), its clinical heterogeneity poses significant diagnostic and prognostic challenges. The disease spectrum ranges from the rapidly fatal childhood cerebral form (CCALD, ~35% of cases) to the chronic adrenomyeloneuropathy (AMN, ~60%) and Addison‐like presentations (Baker et al. 2022; Parasar et al. 2024; Zuo and Chen 2024). This profound variability, particularly the unpredictable progression to lethal cerebral involvement, underscores the critical need for better predictors of disease course and targets for intervention.

The core molecular defect lies in pathogenic variants of the ABCD1 gene, which encodes the peroxisomal membrane protein adrenoleukodystrophy protein (ALDP). ALDP functions as a homodimer essential for transporting VLCFA into peroxisomes for β‐oxidation (Chen et al. 2022; Schleker et al. 2022). ABCD1 mutations disrupt this process, leading to toxic VLCFA accumulation primarily in the adrenal cortex, testes, and neural tissues, driving disease pathogenesis (Chen et al. 2022).

Despite the identification of over 3700 distinct ABCD1 variants cataloged in the X‐ALD Mutation Database (Liu et al. 2022), a fundamental gap persists in understanding the mechanistic relationship between the spectrum of ABCD1 mutations, particularly those disrupting transmembrane and ATP‐binding domains critical for transporter function (Jia et al. 2022; Schleker et al. 2022), and the extreme clinical heterogeneity observed in X‐ALD. This genotype–phenotype discordance severely hinders accurate prognosis and personalized management strategies for affected individuals and families.

In this study, we identified a novel ABCD1 variant (c.773T>G). This variant meets the ACMG criteria for classification as “Likely Pathogenic”. Subsequent functional analyses conclusively demonstrated the pathogenicity of this ABCD1 variant. This finding achieves dual significance: (1) it expands the global ABCD1 pathogenic mutation database, and critically, (2) it provides essential molecular information for accurate clinical genetic diagnosis of X‐ALD patients, particularly within the context of familial genetic counseling.

## 2 Materials and Methods

### 2.1 Ethics Approval and Consent to Participate

This study was approved by the Medical Ethics Committee of SSL, Central Hospital of Dongguan City, Affiliated Dongguan Shilong People's Hospital of Guangdong Medical University. The ethics approval reference number is V1.1 2022–074‐01.

### 2.2 Whole‐Exome Sequencing and Variant Analysis

Genomic DNA was isolated from peripheral blood specimens of clinical subjects using standardized procedures. Blood samples were drawn into EDTA‐coated vacuum collection tubes and processed with the QIAamp DNA Blood Mini Kit (Qiagen) in accordance with the manufacturer's specifications. Comprehensive exome analysis was executed at AmCare Genomic Laboratory (Guangzhou, China) through next‐generation sequencing technology. Target enrichment procedures employed the SureSelectXT HS Human All Exon v7 platform (Agilent Technologies, USA) followed by high‐throughput sequencing on an Illumina NextSeq 550 system (USA).

Raw sequencing data underwent alignment against the GRCh38 human reference assembly through Burrows‐Wheeler Aligner (BWA v0.7.13). Subsequent variant detection and interpretation utilized ANNOVAR computational pipelines integrated with population genomics resources (1000 Genomes, dbSNP, GnomAD) and clinical databases (ClinVar, HGMD, OMIM). Pathogenicity prediction algorithms including SIFT, PolyPhen‐2, Provean, and MutationTaster were systematically applied to assess missense mutation effects. All genetic alterations were classified according to ACMG evidence‐based guidelines for variant interpretation. Familial segregation analysis through bidirectional Sanger sequencing confirmed the inheritance patterns of candidate variants (He et al. 2025).

### 2.3 Vector Construction

The cDNAs encoding ABCD3, wild‐type ABCD1 (ABCD1WT), and the mutant ABCD1 (c.773T>G, ABCD1Leu258Arg) were separately cloned into the pcDNA3.1‐V5 and p3×FLAG‐CMV‐10 expression vectors. All constructs, including p3 × FLAG‐CMV‐10, pcDNA3.1‐V5, p3×FLAG‐CMV‐10‐ABCD1WT, p3×FLAG‐CMV‐10‐ABCD1Leu258Arg, and pcDNA3.1‐V5‐ABCD3, were obtained from Paivi Biosciences Inc. (Wuhan, China). The recombinant plasmids were verified by sequencing to confirm the accuracy of the cloning.

### 2.4 Biochemical Testing and Neuroimaging

VLCFAs were measured using gas chromatography–mass spectrometry (Camtosun et al. 2021). Magnetic resonance imaging (MRI) was employed to identify pathological changes in the brain (Mathkour et al. 2023).

### 2.5 Immunofluorescence Assays

For immunofluorescence, HEK293 cells were initially washed twice with PBS, followed by fixation with 4.0% paraformaldehyde for 10 min. Permeabilization was then performed using 0.1% Triton X‐100 to facilitate antibody penetration. Nonspecific binding sites were blocked by incubating the cells with 5% BSA for 30 min. The cells were subsequently incubated with primary antibodies: anti‐Flag (1:1000, Cell Signaling Technology, #2368) and anti‐V5 (1:1000, Cell Signaling Technology, #80076). Detection was carried out using Alexa 488‐conjugated goat anti‐rabbit (1:400, Abcam, ab150077) and Alexa Cy3‐conjugated goat anti‐mouse (1:400, Abcam, ab97035) secondary antibodies. Nuclei were stained with DAPI (Biosharp, Beijing, China). Imaging was performed using a Leica TCS SP5 confocal microscope equipped with a Plan‐Apochromat 63× NA 1.4 oil immersion differential interference contrast (DIC) objective. Optical sections (0.5 μm thickness) were captured in a *z*‐stack montage (Hu et al. 2023).

### 2.6 Molecular Dynamics Simulation

The *ABCD1* structural topology was acquired from the AlphaFold protein structure database and rendered using the PyMOL molecular visualization system (v3.0.3). Conformational sampling of secondary structure elements was analyzed through PROCHECK's Ramachandran plot validation server (Chauhan et al. 2023). Comparative molecular dynamics (MD) simulations between wild‐type and mutant *ABCD1* conformers were implemented to quantify structural divergence through three principal metrics: backbone root mean square deviation (RMSD), residue‐specific fluctuation (RMSF), and macromolecular compactness (radius of gyration, Rg). Simulation protocols were established using the Gromacs 2022 computational suite (Gruszczyk et al. 2022) with the following parameterization: Amber99sb‐ildn force field application, SPCE explicit water solvation, and cubic periodic boundary conditions (17.695 nm^3^) maintaining a 1.2 nm solute‐box margin (Li et al. 2022). Charge neutralization was achieved through automated ion placement. Energy minimization (50,000‐step steepest descent protocol) preceded system equilibration in dual thermodynamic ensembles: NVT (300 K, V‐rescale thermostat) and NPT (1 bar, Berendsen barostat) (Shino and Takada 2021). Production MD trajectories (100 ns duration) employed Particle Mesh Ewald electrostatics with a 1.0 nm cutoff for both Coulombic and Lennard‐Jones interactions (Wang et al. 2021).

### 2.7 Statistical Analysis

Statistical computations were executed in GraphPad Prism 10 (GraphPad Software, USA) with experimental data expressed as mean ± SEM. Comparative analyses employed parametric hypothesis testing frameworks: two‐tailed Student's *t*‐test for dichotomous comparisons, whereas omnibus one‐way ANOVA with Tukey's multiple comparison test addressed multigroup evaluations. Specific treatment‐control contrasts were resolved using Dunnett's post hoc methodology following ANOVA. All inferential statistics maintained a predetermined alpha level of 0.05 for significance determination.

## 3 Results

### 3.1 Case Description

A 14‐year‐old male patient was admitted to the Neurology Department of Dongguan Songshan Lake Central Hospital due to progressive memory decline over a period of more than 5 months and vision impairment lasting over 4 months. The patient exhibited decreased learning ability, characterized by poor academic performance, slow reactions, recent memory issues, repeatedly asking the same questions, needing frequent reminders for daily activities, and experiencing unclear vision without expressing complaints. Over time, the memory problems worsened, and the patient began to show signs of blurred vision, difficulty reading text on the classroom blackboard, double vision, and distorted visual perception, again without complaints. He was admitted to the hospital for further investigation into the causes of his cognitive and visual impairments. The patient had no significant prior medical history. Given the suspicion of autoimmune encephalitis, he underwent a 5‐day course of high‐dose intravenous immunoglobulin therapy. However, his condition showed no improvement following treatment.

### 3.2 Clinical Diagnosis

**FIGURE 1:**
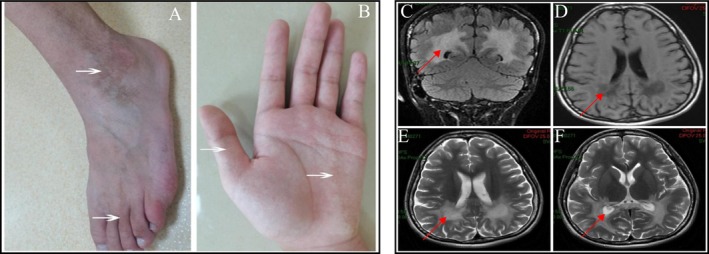
Analysis of skin pigmentation and MRI characteristics in the X‐ALD proband. (A, B) The proband exhibits marked pigmentation in specific areas of the skin. As indicated by the white arrows in the images, significant pigment deposition is observed in the ankles, toes, and palms. (C–F) MRI scans of the proband's brain reveal lesions primarily localized to the white matter surrounding the posterior region (C), posterior horns (D, E), and inferior horns (F) of the bilateral lateral ventricles, with involvement of the corpus callosum. On T1‐weighted imaging (T1WI), these lesions appear as areas of hypointensity, whereas on T2‐weighted imaging (T2WI), they demonstrate hyperintensity. The region denoted by the red arrow demonstrates an abnormal signal within the white matter.

Vital sign evaluation demonstrated normothermic status (36.5°C) with hemodynamic parameters recorded as follows: cardiac rhythm 100 bpm, respiratory cycle frequency 20 breaths/min, and normotensive blood pressure measurement (115/70 mmHg). Notable findings included partial skin hyperpigmentation (Figure 1A,B). No evidence of jaundice, rash, or petechiae was observed. Additionally, no enlargement of superficial lymph nodes was detected upon palpation. Cardiopulmonary and abdominal examinations were unremarkable. The patient exhibited clear and coherent speech. Temporal orientation was intact, while spatial orientation showed mild impairment. Arithmetic ability was tested with the calculation “86 minus 7”, which highlighted mild difficulty. Pupillary examination showed bilaterally equal and round pupils, with intact light reflexes and normal extraocular movements. No nystagmus or other cranial nerve abnormalities were noted. Limb reflexes were increased (++), while bilateral pathological reflexes were absent (−). There was no evidence of meningeal irritation, with negative results for Kernig and Brudzinski signs (−). Cranial MRI demonstrated leukodystrophy affecting the posterior horns of the bilateral lateral ventricles and the peritrigonal white matter, with involvement of the splenium of the corpus callosum, findings consistent with a diagnosis of adrenoleukodystrophy (Figure 1C–F). Electroencephalography (EEG) revealed a borderline pattern. The autoimmune encephalitis autoantibody assay yielded negative results, ruling out autoimmune encephalitis in this patient. The plasma VLCFA profile revealed markedly elevated levels, with C26:0 measured at 5.1 nmol/mL (reference range ≤ 1.3 nmol/mL) and a C26:0/C22:0 ratio of 0.087 (reference range ≤ 0.023) (Table 1). These findings, along with a characteristic peroxisomal profile, confirmed the diagnosis of X‐ALD. Currently, there are no effective therapeutic interventions available for patients with XX‐ALD. Upon diagnosis, hormone replacement therapy is primarily administered to alleviate certain symptoms; however, this treatment does not halt disease progression. The patient's condition typically deteriorates inexorably, with progressive development of various neurological impairments, including progressive cognitive decline, dysarthria, hearing loss, motor dysfunction, urinary incontinence, and visual loss. After a follow‐up period of one and a half years, the patient ultimately succumbed to the disease.

**TABLE 1:** VLCFAs in members of a family with X‐ALD (nmol/mL).

VLCFAs	Proband	Mother of the proband	Father of the proband	Re. value
C22:0	58.5	71.8	60.3	≤ 96.3
C24:0	77.8	73.5	51.9	≤ 91.4
C26:0	5.1↑	0.95	1.05	≤ 1.30
C24:0/C26:0	1.33	1.19	1.08	≤ 1.39
C26:0/C22:0	0.087↑	0.015	0.021	≤ 0.023

### 3.3 Genetic Analysis of X‐ALD Pedigree

**FIGURE 2:**
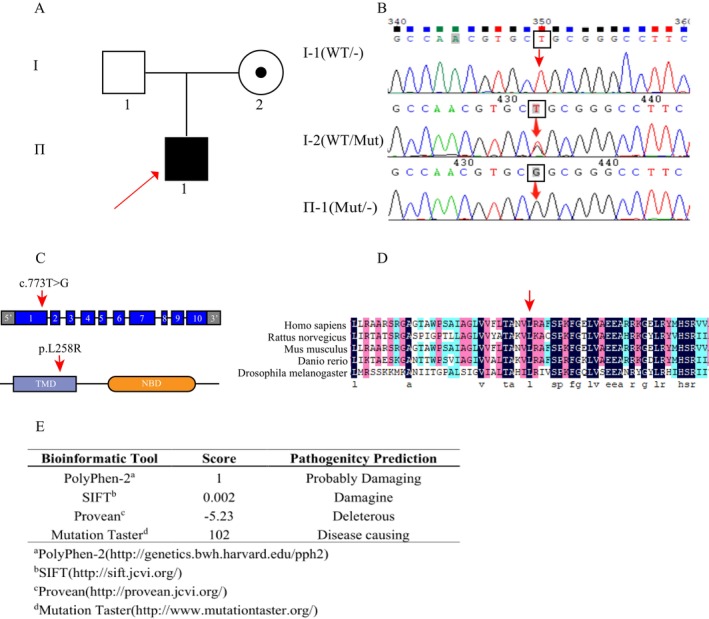
Pedigree analysis of Chinese families with a novel *ABCD1* mutation. (A) The figure illustrates the pedigree of a family affected by X‐linked adrenoleukodystrophy (X‐ALD). Unaffected males are represented by blank squares, female carriers by circles with black dots, and confirmed X‐ALD patients by filled black squares. The proband, identified as a hemizygote, is marked with a red arrow. (B) Sanger sequencing of peripheral blood cDNA samples from the proband (II‐1) and his parents revealed that both the proband and his mother (carrier, I‐2) harbor a novel mutation in the *ABCD1* gene: C.773T>G. This mutation results in a missense change at the protein level, substituting leucine with arginine at position 258 (p.Leu258Arg), and is identified as a newly discovered pathogenic variant. (C) Mutation analysis of the *ABCD1* gene is depicted in two panels. The upper panel provides a schematic representation of the genomic structure of *ABCD1*, which comprises 10 exons (indicated by rectangular boxes). The lower panel illustrates the structural domains of the adrenal leukodystrophy protein (ALDP) encoded by *ABCD1*, including the transmembrane domain (TMD) and the nucleotide‐binding domain (NBD). The red arrow highlights the specific nucleotide alteration and the corresponding site of the amino acid substitution in the ALDP protein. (D) Cross‐species conservation analysis of the *ABCD1* gene sequence reveals that leucine at position 258 (p. Leu258) is highly conserved across multiple species. Following the mutation, leucine is substituted by arginine (p.Leu258Arg), as indicated by the red arrow, underscoring the potential functional significance of this residue. (E) Various bioinformatics tools predict the potential pathogenicity of the *ABCD1* ^p.Leu258Arg^ mutation.

To investigate the molecular etiology of X‐ALD in the familial cohort, comprehensive exome analysis was conducted on the index case (pedigree position II‐1, Figure 2A). This genomic interrogation identified a previously unreported pathogenic variant (c.773T>G; p.Leu258Arg) in the *ABCD1* gene (NM_000033), localized to exon 1. The detected substitution demonstrated complete segregation with the disease phenotype, suggesting its potential causal role in the observed X‐ALD manifestation. This analysis revealed a novel missense mutation, c.773T>G (p.Leu258Arg), in exon 1 of the X‐linked *ABCD1* gene, which appears to be associated with the clinical presentation of X‐ALD in this individual. Sanger sequencing further confirmed the presence of this mutation in both the proband and his mother (Figure 2B), suggesting that the mother is a carrier of the pathogenic *ABCD1* allele. Sequence alignment across various species demonstrated that the affected site is highly conserved (Figure 2D). To assess the pathogenicity of the c.773T>G mutation, bioinformatics tools including Provean, SIFT, PolyPhen‐2, and MutationTaster were employed (Figure 2E). All analyses consistently predicted that this novel mutation in *ABCD1* is indeed pathogenic.

### 3.4 3D Structure Prediction of ALDP

**FIGURE 3:**
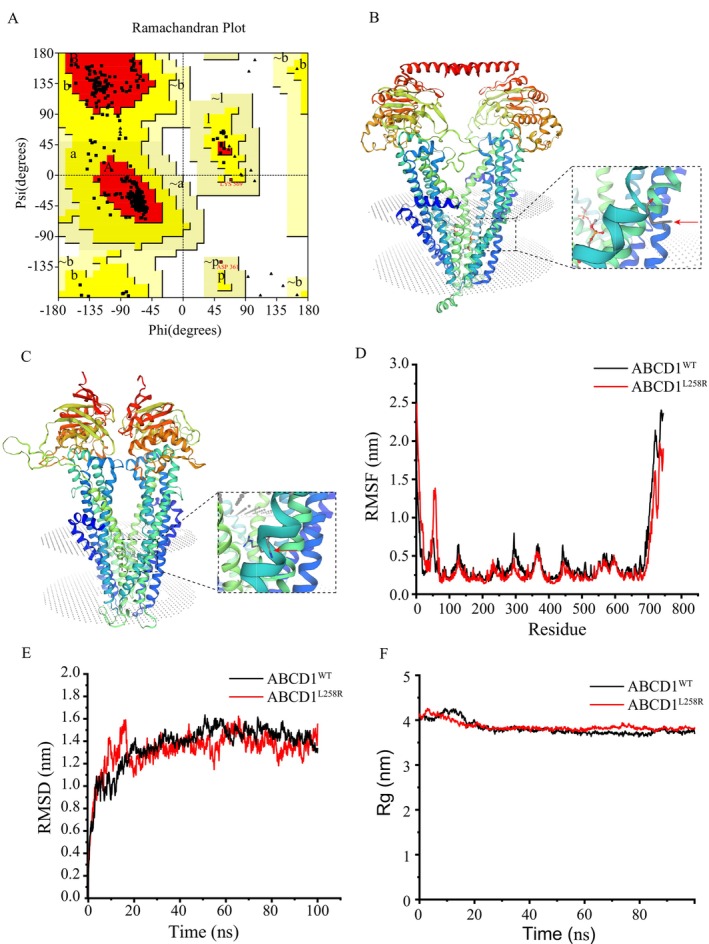
Protein structure and molecular dynamics simulation results of *ABCD1*. (A) Ramachandran diagram of *ABCD1*. (B, C) The 3D computer model (SWISS‐MODEL) predicts the protein structures of normal *ABCD1* and *ABCD1* with p.L258R mutation, and the red arrow shows the substituted amino acids. (D–F) shows the molecular dynamics simulation results of *ABCD1* ^WT^ (black), *ABCD1* ^p.L258R^ (red), RMSF (D), RMSD (E) and Rg (F).

To evaluate the accuracy of the protein structure prediction, this study employed the PROCHECK tool within the SAVESv6.0 toolkit (https://saves.mbi.ucla.edu/) to generate a Ramachandran plot for assessing the stereochemical quality of the predicted model (Agarwal et al. 2023). The results revealed that for the ALDP, 92.8% (1000 residues) of amino acid residues were located in most favored regions, 6.9% (74 residues) in additional allowed regions, and 0.4% (four residues) in generously allowed regions. Importantly, no residues were found in disallowed regions. Notably, LYS569 and ASP361 were positioned in generously allowed regions, suggesting that these residues may constrain the generation of models with improved stereochemical quality (Figure 3A). Computational structural analysis of *ABCD1*‐encoded ALDP was conducted through the SWISS‐MODEL homology modeling platform. This in silico approach enabled systematic evaluation of mutation‐induced conformational alterations in the ALDP tertiary structure (Cheng et al. 2022). Homology modeling, using a template structure from the Protein Data Bank (PDB), was employed to predict the effects of the c.773T>G mutation on ALDP's structure (Figure 3B,C). Literature reports highlight that a high‐density region of missense mutations is concentrated in exons 1 and 2 of *ABCD1* (Zuo and Chen 2024). Further structural analysis revealed that the c.773T>G mutation occurs within exon 1, where the amino acid substitution from leucine 258 to arginine may induce a conformational change in the protein (Figure 2C), potentially disrupting its normal function.

### 3.5 Molecular Dynamics Simulations

To investigate the structural stability and dynamic characteristics of ALDP^WT^ and ALDP^Leu258Arg^ in an aqueous environment, this study performed 100 ns MD simulations. The radius of gyration (Rg) for ALDP^WT^ fluctuated between 3.6 and 4.3 nm, ultimately converging to a stable value of 3.7 nm by the end of the simulation. In contrast, ALDP^Leu258Arg^ exhibited Rg values ranging from 3.73104 to 4.26129 nm, stabilizing at 3.82581 nm at 100 ns (Figure 3F). These results indicate that ALDP^Leu258Arg^ has a slightly reduced compactness compared to ALDP^WT^. The RMSD analysis revealed a rapid increase during the initial phase of the simulation (0–10 ns), corresponding to structural equilibration. After approximately 10 ns, the RMSD values stabilized. For ALDP^WT^, RMSD values fluctuated between 1.2 and 1.5 nm beyond 20 ns, reflecting high structural stability (Figure 3E). The root mean square fluctuation (RMSF) profiles of both proteins exhibited comparable fluctuation patterns across most residues, suggesting similar global flexibility and stability. However, specific regions, including the N‐ and C‐terminal regions as well as residues 53, 128, 295, 367, 442, and 567, showed significantly higher fluctuations in ALDP^Leu258Arg^ relative to ALDP^WT^ (Figure 3D). Increases in flexibility suggest that the p.Leu258Arg mutation induces structural destabilization in these regions. Overall, these findings demonstrate that the p.Leu258Arg mutation compromises the structural stability of ALDP, leading to reduced compactness and increased regional flexibility, which may adversely affect the protein's functional properties.

### 3.6 Subcellular Localization of ALDP^Leu258Arg^ Variant In Vitro

**FIGURE 4:**
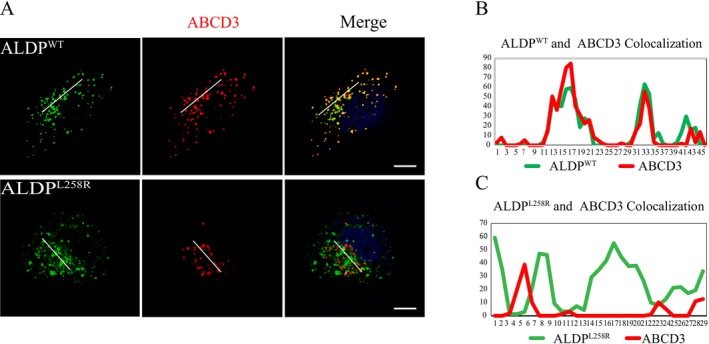
Immunofluorescence Analysis. (A) Human *ABCD1* ^WT^ and mutant *ABCD1* ^Leu258Arg^ expression plasmids were transfected into HEK293 cells to examine the co‐localization of *ABCD1* ^WT^ (green), *ABCD1* ^Leu258Arg^ (green), and *ABCD3* (red) proteins. Nuclei were stained with DAPI (blue). (B, C) The fluorescence intensity distribution of *ABCD1* ^WT^, *ABCD1* ^Leu258Arg^ and *ABCD3* was quantified along a white line drawn through the cells using ImageJ software. Scale bar = 10 μm.

To evaluate whether the Leu258Arg mutation affects the subcellular localization of ALDP, this study utilized immunofluorescence analysis to examine the expression patterns of ALDP^Leu258Arg^ in HEK293 cells. As shown in Figure 4A, all experimental cells displayed fluorescent signals (red) corresponding to the peroxisomal marker *ABCD3*, confirming the presence of intact peroxisomes. In cells expressing ALDP^WT^, the fluorescent signals for ALDP^WT^ (green) co‐localized extensively with those of *ABCD3* (red), resulting in a yellow‐orange signal. This high degree of overlap demonstrates that ALDP^WT^ was correctly targeted to peroxisomes, consistent with accurate expression and functional localization in HEK293 cells (Figure 4B). In contrast, cells expressing ALDP^Leu258Arg^ exhibited a markedly different pattern. The fluorescence signal for ALDP^Leu258Arg^ (green) was predominantly distributed in the cytoplasm, with minimal overlap with the peroxisomal marker *ABCD3* (Figure 4C). This mislocalization suggests that the Leu258Arg mutation disrupts the proper peroxisomal targeting of ALDP. Such aberrant localization could compromise peroxisomal functions, including fatty acid β‐oxidation, by reducing the availability of functional ALDP within the organelle.

## 4 Discussion

X‐ALD is a peroxisomal disorder characterized by neurodegeneration and adrenocortical insufficiency. The core pathogenic mechanism involves loss‐of‐function variants in the *ABCD1* gene, leading to dysfunction of ALDP. This disrupts the peroxisomal transmembrane transport of VLCFA (C22–C26), resulting in their pathological accumulation in plasma and tissues. Global epidemiological studies indicate a male prevalence of approximately 1:20,000, a female prevalence of approximately 1:10,000, and an estimated incidence ranging between 1:20,000 and 1:30,000 (Engelen et al. 2012; Wiesinger et al. 2015).

ALDP belongs to subfamily D of the ATP‐binding cassette (ABC) transporter superfamily (*ABCD*). The human *ABCD* subfamily comprises four members (*ABCD1‐4*), with *ABCD1‐3* localized to the peroxisomal membrane (Tawbeh et al. 2021). These proteins contain conserved transmembrane domains (TMDs) and nucleotide‐binding domains (NBDs), utilizing ATP hydrolysis to drive substrate transport (Janas et al. 2003; Kawaguchi and Imanaka 2022). To date, the X‐ALD Mutation Database (http://www.x‐ald.nl) has cataloged over 3700 *ABCD1* gene variants, predominantly comprising missense mutations (63.8%), nonsense mutations (9.1%), frameshift mutations (15.7%), splice site mutations (4.0%), and exon deletions (2.5%). The vast majority of variants (92.9%) reside within exons, with 6.1% in introns and < 1% in untranslated regions (UTRs) (Janas et al. 2003). Studies demonstrate regional clustering of pathogenic *ABCD1* variants: the TMDs (exons 1–2) harbor the highest proportion (46%), followed by the NBDs (exons 6–9, 35%), with exons 3–4 and 5 accounting for 9% and 7%, respectively (Mallack et al. 2022). Notably, Liu et al. reported that exon 1 represents a mutational hotspot (37.5%) in AMN patients worldwide, with the majority being missense mutations (84.4%) (Zhang et al. 2021). However, a study by Chen et al. on a Chinese cohort (*n* = 14) identified known mutations predominantly within exons 6–9 (9/12), with only a minority in exons 1–2 (3/12), suggesting potential population heterogeneity in mutation distribution and highlighting the importance of larger sample sizes (Zhang et al. 2025).

This study reports a Chinese male adolescent with the cerebral form of X‐ALD (onset at age 14, presenting with progressive cognitive decline and visual/auditory loss). Genetic analysis identified a novel missense mutation in the *ABCD1* gene, c.773T>G (p.Leu258Arg). This variant is located within the known mutational hotspot region of exon 1 and results in the substitution of a highly conserved, nonpolar leucine (Leu) residue at position 258 of the ALDP protein with a polar arginine (Arg). Analysis using variant pathogenicity prediction tools classified this mutation as “Likely Pathogenic”. The variant was absent in 195 healthy controls and public population genomic databases (e.g., gnomAD), excluding it as a common polymorphism. Homology modeling of the protein three‐dimensional structure revealed that the p.Leu258Arg substitution induces a significant conformational change in the *ABCD1* protein. Subcellular localization experiments further demonstrated that while the wild‐type ALDP‐GFP fusion protein co‐localized efficiently with the peroxisomal marker *ABCD3*, co‐localization of the Leu258Arg mutant with *ABCD3* was markedly impaired. Integrating this evidence, in accordance with the ACMG variant interpretation guidelines, we classify c.773T>G (p.Leu258Arg) as a “Pathogenic Variant”.

X‐ALD is classified into seven clinical subtypes based on age of onset and primary organ involvement: childhood cerebral, adolescent cerebral, AMN, adult cerebral, olivo‐ponto‐cerebellar, Addison only, and asymptomatic (Kemp et al. 2001). Cerebral ALD characterizes the pediatric population, whereas AMN primarily affects adults (Kang et al. 2024). Pathogenic variants within exon 1 of *ABCD1* are associated with diverse clinical phenotypes. For instance, patients with p. Pro48Ser, p. Gly277Arg, and p.Ser290Trp variants presented with AMN; those with p.Gln266Arg and p.Ser108Leu exhibited both cerebral and AMN phenotypes; p.Asn148Asp and p.Asn214Asp were associated with the cerebral form; p. Thr254Pro manifested as AMN (Matsukawa et al. 2011). The patient with p.Leu258Arg identified in this study presented with the adolescent cerebral form. These clinical observations further underscore the complexity of genotype–phenotype correlations in *ABCD1*, indicating that variants within exon 1 do not strictly correlate with a single clinical subtype. Phenotypic expression is likely modulated by other genetic or environmental factors.

Overall, this study reports a novel pathogenic missense mutation, c.773T>G (p.Leu258Arg), located within the mutational hotspot exon 1 of the *ABCD1* gene, thereby expanding its mutational spectrum. Through bioinformatic prediction, structural modeling, and functional assays, we demonstrate that this mutation impairs the normal conformation of ALDP and its precise localization to the peroxisomal membrane, ultimately leading to loss of its VLCFA transport function. Our findings advance the understanding of the pathogenic mechanisms underlying *ABCD1* mutations and re‐emphasize the high degree of heterogeneity and complexity in X‐ALD genotype–phenotype relationships. These results provide important experimental evidence for elucidating the molecular pathology of X‐ALD and may contribute to the future development of targeted diagnostic strategies and therapies.

## Author Contributions

Designed the study: J.H., P.H., and H.F.; Performed experiments: H.F., L.H., X.L., and B.H.; Analyzed the data: J.H. and P.H.; Drafted the manuscript: J.H. and P.H. All authors read and approved the final manuscript.

## Conflicts of Interest

The authors declare no conflicts of interest.

## Data Availability

The data that support the findings of this study are available on request from the corresponding author. The data are not publicly available due to privacy or ethical restrictions.

## References

[mgg370148-bib-0001] Agarwal, D. , G. Kumar , M. Ashraf Rather , and I. Ahmad . 2023. “Cloning, Computational Analysis and Expression Profiling of Steroid 5 Alpha‐Reductase 1 (SRD5A1) Gene During Reproductive Phases and Ovatide Stimulation in Endangered Catfish, Clarias Magur.” Scientific Reports 13: 19553. 37945678 10.1038/s41598-023-46969-1 PMC10636143

[mgg370148-bib-0002] Baker, C. V. , A. Cady Keller , R. Lutz , et al. 2022. “Newborn Screening for X‐Linked Adrenoleukodystrophy in Nebraska: Initial Experiences and Challenges.” International Journal of Neonatal Screening 8: 29. 35645283 10.3390/ijns8020029 PMC9149921

[mgg370148-bib-0003] Camtosun, E. , I. Dundar , A. Akinci , L. Kayas , and N. Ciftci . 2021. “Pediatric Primary Adrenal Insufficiency: A 21‐Year Single Center Experience.” Journal of Clinical Research in Pediatric Endocrinology 13: 88–99. 32938577 10.4274/jcrpe.galenos.2020.2020.0132 PMC7947721

[mgg370148-bib-0004] Chauhan, R. , A. Gupta , L. Malhotra , et al. 2023. “Ubiquitin Specific Peptidase 37 and PCNA Interaction Promotes Osteosarcoma Pathogenesis by Modulating Replication Fork Progression.” Journal of Translational Medicine 21: 286. 37118828 10.1186/s12967-023-04126-2 PMC10142227

[mgg370148-bib-0005] Chen, H. A. , R. H. Hsu , P. W. Chen , et al. 2022. “High Incidence of Null Variants Identified From Newborn Screening of X‐Linked Adrenoleukodystrophy in Taiwan.” Molecular Genetics and Metabolism Reports 32: 100902. 36046390 10.1016/j.ymgmr.2022.100902 PMC9421440

[mgg370148-bib-0006] Cheng, L. , Y. Li , W. Zhou , and T. Bo . 2022. “Case Report: Novel Mutation of F5 With Maternal Uniparental Disomy Causes Severe Congenital Factor V Deficiency.” Frontiers in Pediatrics 10: 913050. 35747490 10.3389/fped.2022.913050 PMC9211043

[mgg370148-bib-0007] Engelen, M. , S. Kemp , M. de Visser , et al. 2012. “X‐Linked Adrenoleukodystrophy (X‐ALD): Clinical Presentation and Guidelines for Diagnosis, Follow‐Up and Management.” Orphanet Journal of Rare Diseases 7: 51. 22889154 10.1186/1750-1172-7-51 PMC3503704

[mgg370148-bib-0008] Gruszczyk, J. , L. Grandvuillemin , J. Lai‐Kee‐Him , et al. 2022. “Cryo‐EM Structure of the Agonist‐Bound Hsp90‐XAP2‐AHR Cytosolic Complex.” Nature Communications 13: 7010. 10.1038/s41467-022-34773-w PMC9668932 36385050

[mgg370148-bib-0009] He, P. , S. Liu , X. Shi , et al. 2025. “A Novel Homozygous Missense ZP1 Variant Result in Human Female Empty Follicle Syndrome.” Clinical Genetics 107: 147–156. 39380244 10.1111/cge.14624

[mgg370148-bib-0010] Hu, J. , X. Guan , M. Zhao , P. Xie , J. Guo , and J. Tan . 2023. “Genome‐Wide CRISPR‐Cas9 Knockout Screening Reveals a TSPAN3‐Mediated Endo‐Lysosome Pathway Regulating the Degradation of Alpha‐Synuclein Oligomers.” Molecular Neurobiology 60: 6731–6747. 37477766 10.1007/s12035-023-03495-5

[mgg370148-bib-0011] Janas, E. , M. Hofacker , M. Chen , S. Gompf , C. van der Does , and R. Tampe . 2003. “The ATP Hydrolysis Cycle of the Nucleotide‐Binding Domain of the Mitochondrial ATP‐Binding Cassette Transporter Mdl1p.” Journal of Biological Chemistry 278: 26862–26869. 12746444 10.1074/jbc.M301227200

[mgg370148-bib-0012] Jia, Y. , Y. Zhang , W. Wang , J. Lei , Z. Ying , and G. Yang . 2022. “Structural and Functional Insights of the Human Peroxisomal ABC Transporter ALDP.” eLife 11: e75039. 36374178 10.7554/eLife.75039 PMC9683791

[mgg370148-bib-0013] Kang, Y. , L. Guo , Z. Min , L. Zhang , L. Zhang , and C. Tang . 2024. “Brainstem Dominant Form of X‐Linked Adrenoleukodystrophy With a Novel ABCD1 Missense Variant: A Case Report and Literature Review.” Molecular Genetics & Genomic Medicine 12: e2499. 39051462 10.1002/mgg3.2499 PMC11270050

[mgg370148-bib-0014] Kawaguchi, K. , and T. Imanaka . 2022. “Substrate Specificity and the Direction of Transport in the ABC Transporters ABCD1‐3 and ABCD4.” Chemical & Pharmaceutical Bulletin 70: 533–539. 35908918 10.1248/cpb.c21-01021

[mgg370148-bib-0015] Kemp, S. , A. Pujol , H. R. Waterham , et al. 2001. “ABCD1 Mutations and the X‐Linked Adrenoleukodystrophy Mutation Database: Role in Diagnosis and Clinical Correlations.” Human Mutation 18: 499–515. 11748843 10.1002/humu.1227

[mgg370148-bib-0016] Li, J. , Q. Wang , and Y. Tu . 2022. “Binding Modes of Prothrombin Cleavage Site Sequences to the Factor Xa Catalytic Triad: Insights From Atomistic Simulations.” Computational and Structural Biotechnology Journal 20: 5401–5408. 36212544 10.1016/j.csbj.2022.09.030 PMC9529552

[mgg370148-bib-0017] Liu, S. , L. Li , H. Wu , et al. 2022. “Genetic Analysis and Prenatal Diagnosis of 76 Chinese Families With X‐Linked Adrenoleukodystrophy.” Molecular Genetics & Genomic Medicine 10: e1844. 34826210 10.1002/mgg3.1844 PMC8801145

[mgg370148-bib-0018] Mallack, E. J. , K. Gao , M. Engelen , and S. Kemp . 2022. “Structure and Function of the ABCD1 Variant Database: 20 Years, 940 Pathogenic Variants, and 3400 Cases of Adrenoleukodystrophy.” Cells 11: 283. 35053399 10.3390/cells11020283 PMC8773697

[mgg370148-bib-0019] Mathkour, M. , C. D. Werner , R. F. Dallapiazza , et al. 2023. “Endoscopically‐Assisted Percutaneous Trigeminal Rhizotomy for Trigeminal Neuralgia: A Cadaveric Feasibility Study.” Asian Journal of Neurosurgery 18: 40–44. 37056893 10.1055/s-0043-1761230 PMC10089747

[mgg370148-bib-0020] Matsukawa, T. , M. Asheuer , Y. Takahashi , et al. 2011. “Identification of Novel SNPs of ABCD1, ABCD2, ABCD3, and ABCD4 Genes in Patients With X‐Linked Adrenoleukodystrophy (ALD) Based on Comprehensive Resequencing and Association Studies With ALD Phenotypes.” Neurogenetics 12: 41–50. 20661612 10.1007/s10048-010-0253-6 PMC3029816

[mgg370148-bib-0021] Mukherjee, D. , P. Sarkar , A. Pandit , B. K. Ray , G. Das , and S. Dubey . 2024. “A Spectrum of Cognitive‐Behavioral‐Movement Disorders in Adrenoleukodystrophy: A Case Series From a Tertiary Care Centre in the Eastern Part of India.” Qatar Medical Journal 2024: 43. 39376208 10.5339/qmj.2024.43 PMC11456738

[mgg370148-bib-0022] Parasar, P. , N. Kaur , and J. Singh . 2024. “Pathophysiology of X‐Linked Adrenoleukodystrophy: Updates on Molecular Mechanisms.” Journal of Biotechnology and Biomedicine 7: 277–288. 39056013 10.26502/jbb.2642-91280151 PMC11271253

[mgg370148-bib-0023] Schleker, E. S. M. , S. Buschmann , H. Xie , S. Welsch , H. Michel , and C. Reinhart . 2022. “Structural and Functional Investigation of ABC Transporter STE6‐2p From Pichia Pastoris Reveals Unexpected Interaction With Sterol Molecules.” Proceedings of the National Academy of Sciences of the United States of America 119: e2202822119. 36256814 10.1073/pnas.2202822119 PMC9618074

[mgg370148-bib-0024] Shino, G. , and S. Takada . 2021. “Modeling DNA Opening in the Eukaryotic Transcription Initiation Complexes via Coarse‐Grained Models.” Frontiers in Molecular Biosciences 8: 772486. 34869598 10.3389/fmolb.2021.772486 PMC8636136

[mgg370148-bib-0025] Tawbeh, A. , C. Gondcaille , D. Trompier , and S. Savary . 2021. “Peroxisomal ABC Transporters: An Update.” International Journal of Molecular Sciences 22: 6093. 34198763 10.3390/ijms22116093 PMC8201181

[mgg370148-bib-0026] Wang, Q. , J. Yang , Z. Zhong , J. A. Vanegas , X. Gao , and A. B. Kolomeisky . 2021. “A General Theoretical Framework to Design Base Editors With Reduced Bystander Effects.” Nature Communications 12: 6529. 10.1038/s41467-021-26789-5 PMC8586357 34764246

[mgg370148-bib-0027] Wiesinger, C. , F. S. Eichler , and J. Berger . 2015. “The Genetic Landscape of X‐Linked Adrenoleukodystrophy: Inheritance, Mutations, Modifier Genes, and Diagnosis.” Application of Clinical Genetics 8: 109–121. 25999754 10.2147/TACG.S49590 PMC4427263

[mgg370148-bib-0028] Zhang, Y. , X. Shi , J. Huang , et al. 2025. “Genotypic and Phenotypic Characteristics of Pediatric X‐Adrenoleukodystrophy in a Chinese Cohort.” Neuropsychiatric Disease and Treatment 21: 677–687. 40165924 10.2147/NDT.S507632 PMC11956711

[mgg370148-bib-0029] Zhang, Y. , G. Zhang , W. Chen , et al. 2021. “A Novel ABCD1 G1202A Mutation in a Chinese Patient With Pure Adrenomyeloneuropathy and Literature Review.” Genes & Diseases 8: 709–714. 34291142 10.1016/j.gendis.2020.01.009 PMC8278541

[mgg370148-bib-0030] Zuo, X. , and Z. Chen . 2024. “From Gene to Therapy: A Review of Deciphering the Role of ABCD1 in Combating X‐Linked Adrenoleukodystrophy.” Lipids in Health and Disease 23: 369. 39529100 10.1186/s12944-024-02361-0 PMC11552335

